# Cognitive Age Delta as a Marker of Healthy and Pathological Cognitive Aging: The Role of Lifestyle, Cognitive Reserve, and Vascular Risk

**DOI:** 10.3390/jcm14228176

**Published:** 2025-11-18

**Authors:** Ainara Estanga, Iñigo Tellaetxe-Elorriaga, Mirian Ecay-Torres, Jorge García Condado, Maite García-Sebastián, Maria Arriba, Carolina López, Naia Ros, Ane Iriondo, Imanol Reparaz-Escudero, Asier Erramuzpe, Pablo Martínez-Lage, Miren Altuna

**Affiliations:** 1 Center for Research and Memory Clinic, CITA-Alzheimer Foundation, 20009 Donostia-San Sebastián, Spain; 2 Computational Neuroimaging Laboratory, Biobizkaia Health Research Institute, 48903 Barakaldo, Spain; 3 Biomedical Research Doctorate Program, Universidad Del País Vasco (EHU), 48940 Leioa, Spain; 4 Department of Clinical and Health Psychology and Research Methods, University of the Basque Country UPV/EHU, 20018 Donostia-San Sebastián, Spain; 5 Navarrabiomed, Hospital Universitario de Navarra (HUN), Universidad Pública de Navarra (UPNA), IdiSNA, 31008 Pamplona, Spain; 6 Ikerbasque, the Basque Foundation for Science, 48009 Bilbao, Spain; 7 Department of Neurology, Bioaraba Health Research Institute, Araba University Hospital-Txagorritxu, 01009 Vitoria-Gasteiz, Spain

**Keywords:** aging, cognition, age modeling, cognitive age delta, cognitive reserve, vocabulary, lifestyle factors, Alzheimer’s disease, biomarkers, vascular pathology

## Abstract

**Background:** Chronological age is an imprecise proxy for cognitive aging. The Cognitive Age Delta (CAD)—the difference between predicted cognitive age and chronological age—offers a scalable, individualized marker of functional brain aging. We examined determinants of CAD in cognitively unimpaired (CU) adults stratified by Alzheimer’s disease (AD) and vascular biomarkers. **Methods**: We analyzed 177 CU participants from the Gipuzkoa Alzheimer Project (Basque Country, Northern Spain) classified as amyloid-negative/vascular-negative (CUA−V−, n = 140), amyloid-positive (CUA+, n = 23), or vascular-positive (CUV+, n = 14) using CSF and MRI criteria; vascular burden was defined as Fazekas ≥ 2 on T2-FLAIR or ≥4 microbleeds on SWI, excluding non-traumatic superficial siderosis and established ischemic lesions. MRI was used solely for vascular classification. Associations with demographic, genetic, lifestyle, and reserve measures were tested with General Linear Models. **Results**: CAD did not differ across biomarker groups (Kruskal–Wallis H(2) = 0.17, *p* = 0.91). Median (IQR) CAD values were 0.28 (−4.13, 4.69) for CUA−V−, −0.14 (−3.15, 2.87) for CUA+, and 0.77 (−2.22, 3.76) for CUV+, indicating comparable distributions. Higher vocabulary scores (proxy of cognitive reserve) related to a younger cognitive age in CUA−V− (β = −1.39, *p* < 0.001) and CUA+ (β = −2.08, *p* = 0.054). In CUA+, greater sedentary time—particularly computer-based sitting—was also associated with lower CAD (daily sitting β = −2.13, *p* = 0.009; workday computer sitting β = −2.32, *p* = 0.015). CAD showed no associations with CSF Aβ42, p-tau or t-tau, APOE ε4 load, or vascular risk factors (all *p* > 0.05). **Conclusions:** CAD captures interindividual resilience-related variability beyond classical AD biomarkers. Vocabulary, a marker of lifelong enrichment, emerged as a robust determinant of a younger cognitive age, while amyloid and vascular pathology exerted limited influence at preclinical stages. These findings support CAD as a sensitive, scalable endpoint for identifying protective factors and guiding personalized prevention in early Aging.

## 1. Introduction

The global increase in life expectancy is reshaping demographic structures worldwide, leading to a rapid growth in the proportion of older adults. This demographic shift is accompanied by a parallel rise in the prevalence of age-related cognitive decline and dementia, which represent major medical, social, and economic challenges [1]. Cognitive impairment not only diminishes quality of life and independence but also imposes an enormous burden on healthcare systems and caregivers. Consequently, the promotion of healthy cognitive aging and early detection of individuals at risk have become critical priorities for brain health initiatives in neurology and geriatrics.

There is increasing recognition that interventions targeting the earliest, ideally in preclinical phases of disease are likely to be the most effective for prevention. In this context, evaluating the impact of established risk factors, both biological (e.g., APOE genotype, amyloid pathology, vascular cerebral lesions) [2] and potentially modifiable behaviors (e.g., physical activity, sleep, diet, cognitive and social engagement) in cognitively unimpaired (CU) individuals is essential [1,3,4]. Such an approach enables us to disentangle mechanisms of healthy cognitive aging from preclinical disease processes and to identify determinants that accelerate or protect against decline, even before clinical symptoms emerge.

Reliance on chronological age as a measure of cognitive aging is limited because it does not capture the substantial interindividual variability in cognitive trajectories. Cognitive aging is a heterogeneous process, with domain-specific differences in memory, executive function, attention, and processing speed [5]. This variability is shaped by complex interactions among biology, lifestyle, and cognitive reserve or resilience [6,7]. Hence, there is a strong rationale for developing individualized, functional markers of cognitive aging that can go beyond chronological age and have the potential to detect subtle deviations earlier that would be apparent clinically.

One promising strategy is to adapt brain age models widely used in neuroimaging to the neuropsychological domain. Recently, regression-based approaches have been developed using brief cognitive test batteries to predict an individual’s cognitive age [8]. The difference between predicted cognitive age and actual chronological age, the Cognitive Age Delta (CAD), reflects whether a person’s cognitive performance is “older” or “younger” than expected. CAD has already shown clinical relevance: in mild cognitive impairment, higher CAD predicted progression to Alzheimer’s disease (AD), demonstrating that short neuropsychological assessments can yield a powerful, scalable biomarker [8]. Thus, cognitive screening tools, beyond their traditional diagnostic role, may provide added value as surrogate markers of functional brain aging.

The importance of CAD is further underscored by the shift toward biomarker-based definitions of AD. According to the most recent research framework, AD is defined strictly by amyloid and/or tau biomarkers, even in asymptomatic individuals [9,10]. At the same time, longitudinal evidence shows that much of the decline traditionally attributed to healthy aging is actually driven by undetected AD pathology or cerebrovascular lesions [11,12,13]. Examining CU older adults who are AD biomarker-negative defined by ATN system (A-) and cerebral vascular burden negative (V-) thus provides a unique opportunity to study genuine healthy cognitive aging largely unconfounded by covert pathology. This approach enhances the internal validity of CAD as a marker of cognitive aging and aligns with prevention-oriented research.

Beyond pathology, lifestyle and psychosocial factors are critical, modifiable targets for promoting late-life cognitive health [1]. Meta-analysis and large cohort studies consistently show that regular physical activity, healthy diet, adequate sleep, abstinence from smoking, moderation in alcohol use, and sustained intellectual and social engagement are associated with better cognitive outcomes and lower risk of dementia [6,14,15,16,17]. Nevertheless, most of this evidence relates to clinical outcomes such as incident dementia or average decline at the population level. Much less is known about how these exposures influence the individualized CAD metric in biomarker-defined CU populations. Identifying factors associated with a younger CAD could highlight actionable targets for primary prevention and provide mechanistic insight into resilience.

The present study builds on this framework by applying CAD modeling to a well-characterized cohort of cognitively unimpaired older adults. We first examine CU A-/V- individuals to characterize determinants of cognitive aging in the absence of amyloid and significant vascular pathology. In parallel, we extend analyses to CU participants stratified by distinct biological risk profiles, specifically those with amyloid positivity only (CUA+) and vascular positivity only (CUV+). This stratification allows us to test whether the same lifestyle and psychosocial factors modulate CAD across groups with distinct biological vulnerabilities.

Our overarching aim is twofold: first, to establish CAD as a sensitive, added-value marker of cognitive function in the biomarker era; and second, to identify modifiable lifestyle and psychosocial determinants of CAD across both pathology-free and pathology-positive CU individuals. By pinpointing factors that consistently relate to a younger cognitive age, we seek to highlight targets for personalized prevention strategies and provide novel insights into resilience mechanisms in aging.

## 2. Materials and Methods

### 2.1. Participants and Design

This cross-sectional study was conducted within the Gipuzkoa Alzheimer Project (GAP), led by CITA-Alzheimer Foundation, a longitudinal cohort on preclinical and prodromal phases of AD in the Basque Country (Northern Spain). Participants were recruited between 2011–2013 from the community through local media advertisements and presentations at the local Alzheimer’s Association (AFAGI—Alzheimer Gipuzkoa). The cohort enrolls 411 adults aged 40–80 years without dementia and without neurological, psychiatric or systemic conditions that could affect cognition. The inclusion/exclusion criteria and study procedures have been fully described earlier [18,19]. Each participant underwent a comprehensive clinical, neurological, and neuropsychological evaluation, complemented by assessments of diet and lifestyle factors, as well as structural magnetic resonance imaging (MRI) and biological sampling, and optional cerebrospinal fluid (CSF) collection by lumbar puncture. To minimize potential desynchronization across modalities, the neurological examination, neuropsychological testing, nursing/vascular measurements, and lifestyle data collection (nutrition and physical activity) were completed across two onsite visits scheduled in two consecutive weeks. For participants who consented to lumbar puncture, CSF collection occurred on average 3.92 months after the baseline visits (SD = 4.23 months), ensuring that all variables analyzed in the present study refer to the same baseline time window.

The study was conducted in accordance with international ethical recommendations for medical research in humans, adhering to the standards of the Declaration of Helsinki (1964 and subsequent revisions) and applicable Spanish legislation. The protocol, informed consent form, and all required documentation were reviewed and approved by the Clinical Research Ethics Committee of the Health Area of Gipuzkoa (approval date: 20 October 2010). Written informed consent was obtained from all participants prior to enrollment. Data confidentiality was strictly ensured in compliance with Organic Law 3/2018, of 5 December, on Data Protection and Digital Rights Guarantee; all files were stored in an independent, encrypted database.

### 2.2. Clinical and Neurological Evaluation

Participants underwent a standardized clinical and neurological evaluation that included medical history, systemic and neurological examination, and family history of dementia, including the degree of relatedness (first vs. non–first degree relative) and the dementia subtype, when possible. Motor performance was examined with the Unified Parkinson’s Disease Rating Scale motor section (UPDRS-III) and the Tinetti Gait and Balance scales [20]. Functional independence was measured with the Assessment of Instrumental Activities of Daily Living (ADCS-ADL) [21], and subjective cognitive complaints were recorded through structured interviews and the Memory Failures of Everyday Questionnaire (MFE) [22]. Neuropsychiatric and affective symptoms were evaluated using the Neuropsychiatric Inventory (NPI) [23] and the Hospital Anxiety and Depression Scale (HADS) [24]. Subjective sleep quality and perceived stress were assessed with the Pittsburgh Sleep Quality Index (PSQI) [25] and the Perceived Stress Questionnaire (PSQ) [26], respectively.

Vascular and anthropometric parameters were defined according to ENRICA criteria [27], including metabolic syndrome, obesity (based on body mass index (BMI) and waist-to-hip ratio), hypertension, diabetes, hypercholesterolemia (total, LDL, HDL), and hypertriglyceridemia. Systolic and diastolic blood pressure, heart rate, and right and left ankle–brachial index were also recorded. Anthropometric parameters included body mass index and waist-to-hip ratio. Blood biochemistry was analyzed under fasting conditions and included the following parameters: total cholesterol (mg/dL), HDL cholesterol (mg/dL), LDL cholesterol (mg/dL), triglycerides (mg/dL), C-reactive protein (mg/L), fasting glucose (mg/dL), fasting insulin (µU/mL), glycated hemoglobin (HbA1c, in both IFCC mmol/mol and NGSP % units), vitamin B12 (pg/mL), and folate (ng/mL).

### 2.3. Neuropsychological Assessment

A comprehensive neuropsychological battery was administered by trained neuropsychologists. The assessment covered global cognition and the major cognitive domains of memory, language, visuospatial ability, attention, processing speed, and executive function. Global cognition was measured with the Mini-Mental State Examination (MMSE) [28] and Memory Alteration Test (M@T) [29]. Memory was assessed using the Free and Cued Selective Reminding test (FCSRT) [30,31] and the Rey–Osterrieth Complex Figure Test (ROCF) immediate and delayed recall [31,32]. Language abilities were evaluated with the Boston Naming Test (BNT) [33,34] and verbal fluency tasks, including both semantic fluency (“animals”) and phonemic fluency (letter “P”) [35]. Visuospatial and visuoconstructive skills were examined using the Rey–Osterrieth Complex Figure copy [31,32], Judgement of Line Orientation (JLO) [36] and the 15-Objects Test [37]. Attention and processing speed were assessed with the Digit Symbol–Coding subtest from the Wechsler Adult Intelligence Scale, 3rd edition (WAIS-III) [38], Digits from the WAIS-III [38] and the Trail Making Test Part A (TMT-A) [39]. Executive functioning was measured using the Trail Making Test Part B (TMT-B) [39], the Stroop Word–Color Interference Test [40] and Letter and Number sequencing subtest from the WAIS-III [38]. All tests were administered according to standardized procedures, and raw scores were interpreted using validated normative data adjusted for age, sex, and education level [41].

### 2.4. Cognitive Reserve

Premorbid intellectual functioning was estimated using the Vocabulary subtest from WAIS-III. Cognitive reserve was further assessed with the Cognitive Reserve Questionnaire [42], developed to quantify experiences contributing to cognitive reserve such as years of formal education, occupational attainment, engagement in cognitively stimulating activities (e.g., reading, playing musical instruments, learning languages, playing chess). Engagement in everyday activities was quantified with the Leisure and Productive Activities Questionnaire [43], which summarizes intellectual, cultural and social activities (e.g., reading, theatre, meeting friends, travelling) and productive roles (e.g., childcare/eldercare, crafts, housework, shopping).

### 2.5. Lifestyle Variables

Lifestyle-related evaluation included physical activity through total score from the IPAQ questionnaire [44], and sedentary behavior assessed with a questionnaire from the “Seguimiento Universidad de Navarra” (SUN) Study (1999), which records average daily hours spent in different sedentary contexts (television, computer use, driving, and other activities sitting or lying down) on weekdays and weekends. In the present analyses, we focused on total sitting time, computer-based sitting, and time spent driving. Dietary habits (Mediterranean diet adherence), tobacco use (active smoker status and number of cigarettes per day), and alcohol consumption (weekly and daily alcohol units) were also collected. 

### 2.6. Syndromic Cognitive Diagnosis

Syndromic cognitive diagnosis was established through consensus meetings between neurologists and neuropsychologists. Mild Cognitive Impairment (MCI) was defined as objective dysfunction using age, sex and education adjusted norms, in at least one cognitive domain together with subjective cognitive decline reported by the participant, a study partner, or the clinician. CU status required no objective cognitive impairment across domains and preserved functional independence. Participants with subjective cognitive complaints alone or isolated low performance on a single test, in the absence of subjective cognitive decline that did not meet impairment criteria were classified as CU.

### 2.7. Genetic Risk

Genotyping of APOE polymorphisms was performed on DNA extracted from peripheral blood leukocytes using polymerase chain reaction (PCR) amplification following established protocols [44]. Participants were classified into three categories according to APOE ε4 allele load: homozygotes (ε4/ε4), heterozygotes (one ε4), and non-carriers (no ε4 allele).

### 2.8. CSF AD Biomarker

CSF samples were obtained and collected under aseptic conditions using an atraumatic needle and without sedation, following international consensus recommendations as described previously [45]. Concentrations of amyloid-β_42_ (Aβ_42_), total tau (t-tau), and phosphorylated tau (p-tau) were quantified using INNOTEST^®^ enzyme-linked immunosorbent assays (ELISA; Fujirebio Europe, Ghent, Belgium) according to the manufacturer’s instructions. All samples were analyzed at the Clinical Neurochemistry Laboratory of Hospital Sant Pau (Barcelona, Spain).

CSF amyloid and tau biomarkers were interpreted using the cutoff values established by Alcolea et al. [46]: Aβ_42_ < 550 pg/mL (A+), p-tau > 61 pg/mL (T+), and t-tau > 350 pg/mL (N+). For Aβ_42_, the threshold was adjusted to 580 pg/mL by adding a 5% confidence interval following previously described approach [47]. Based on these criteria, amyloid (A), tau pathology (T), and neurodegeneration (N) status were determined, and participants were categorized within the AT(N) framework [9,10,48].

### 2.9. MRI Vascular Pathology

MRI was acquired on a Siemens Magnetom Trio Tim 3T scanner. White matter hyperintensities (WMH) were assessed on axial T2-weighted FLAIR sequences, and cerebrovascular burden was defined by the presence of severe WMH (Fazekas score ≥ 2) [49], or four or more cerebral microbleeds (CMBs) identified on susceptibility-weighted imaging (SWI) sequences. In addition, participants presenting with cortical superficial siderosis (cSS) of non-traumatic origin or established ischemic lesions (either lacunar infarcts or territorial infarctions) were excluded from the analyses to minimize confounding effects of overt cerebrovascular injury.

MRI data were used exclusively to define vascular burden (V+), not to classify neurodegeneration (N+), given the limited sensitivity and specificity of structural MRI, particularly visual atrophy scales such as the Scheltens’ Medial Temporal Atrophy (MTA) scale, for detecting early AD related neurodegeneration in CU individuals [50]. This methodological decision is consistent with the recent biological definitions of Alzheimer’s disease proposed by Jack et al. [10] (NIA-AA) and Dubois et al. [9] (IWG-3), which no longer require the presence of neurodegeneration (N+) for the definition of AD, acknowledging that such changes typically occur at more advanced stages and that MRI-based atrophy markers are non-specific at the preclinical phase.

This operational definition of vascular positivity (V+) adheres to current STRIVE and STRIVE-2 recommendations [51,52] and mirrors the neuroimaging exclusion criteria applied in pivotal anti-amyloid monoclonal antibody trials (e.g., Lecanemab, Donanemab), where similar thresholds for CMBs and siderosis are used to identify individuals with vascular fragility and increased susceptibility to amyloid-related imaging abnormalities (ARIA) [53,54]. This alignment ensures both biological validity and clinical relevance of the V+ construct within the context of preclinical AD.

### 2.10. Biomarker Defined Classification of CU Participants

**Figure 1:**
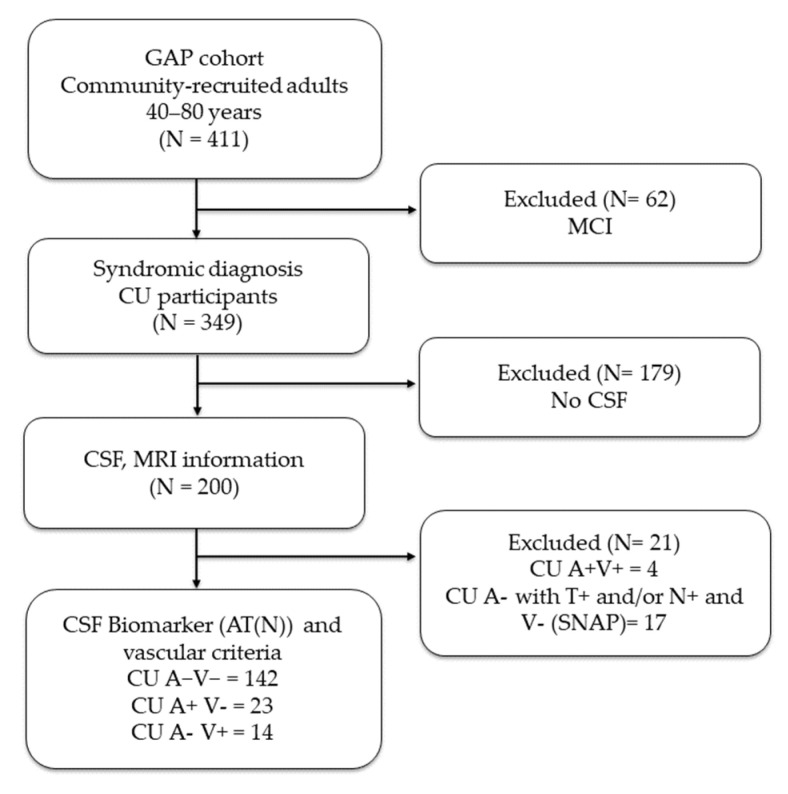
Participant Flow Diagram CAD Study. Abbreviations: MCI = Mild Cognitive Impairment; CU = Cognitively Unimpaired; CSF = Cerebrospinal fluid; GAP = Gipuzkoa Alzheimer Project; A = Amyloid pathology; V = Vascular pathology.

For the present study, analyses were restricted to CU individuals with available CSF and MRI data, enabling the examination of cognitive aging in both pathology-free and pathology-positive participants. This approach better isolates age-related variability from early neurodegenerative effects, and helps minimize potential circularity, as neuropsychological test scores contribute to both diagnostic adjudication and the features used in the CAD model. The participant selection process is illustrated in Figure 1.

Participants were subsequently assigned to one of three CU subgroups based on the A/T/N classification and cerebrovascular burden:

1. CU Biomarker negative (CUA-V-) (n = 142): A−T−N− and V−.
2. CU Amyloid pathology (CUA+) (n = 23): A+ (irrespective of T, N) and V-.
3. CU Vascular pathology (CUV+) (n = 14): V+ with otherwise biomarker-negative ATN profile (A−T−N−).

### 2.11. Cognitive Age Modeling and CAD

Feature preselection was guided by the association between chronological age and individual neuropsychological test scores within the CUA-V- group to identify age-sensitive measures. Specifically, we computed the Pearson correlation coefficients between chronological age and all primary scores from the neuropsychological battery covering global cognition and major domains. Features with correlation coefficients passing Bonferroni multiple comparisons correction were included in the model, trying to keep the number of features to the minimum and the widest cognitive domain representation, while prioritizing the highest correlation coefficients. Highly collinear ρ > 0.7 features were also discarded to mitigate the noise in the model. The final selection of features includes: Digit Symbol-Coding (WAIS-III) subtest, Stroop Color Naming Test, Stroop Word-Color Interference Test, 15-Objects Test, ROCF Delayed Recall, TMT-A, FCSRT Immediate Total Free Recall, TMT-B, Boston Naming Test, Phonemic Verbal Fluency Test (“P”), FCSRT Immediate Total Recall, Semantic Verbal Fluency Test (“Animals”).

The features were corrected for education years to remove the well-known confounding effect of this variable in neuropsychological test performances [41,55,56] and to prevent it from leaking into the subsequent association analyses. This was done first estimating the effect of the education years, sex, and APOE4 allele load [57,58,59] in the CADs with a General Linear Model (GLM) (CAD ~ education years + sex + APOE4 load) to also identify other potential covariates of interest. We found a moderate negative effect between education years and CADs (=−0.99, *p* = 2.09 × 10^−4^) but not with sex (=0.01, *p* = 0.98) nor APOE4 allele load (=0.03, *p* = 0.91). Then, the residuals of a linear model estimating the score using the years of education were added to the original mean of the scores to maintain their scale and ranges, as shown in Equation (1):

(1) $$X_{corrected}=\bar{X}+\hat{\beta }\left(X-\hat{X}\right)$$

where:

$\bar{X}$ = mean of the original scores

$\hat{X}$ = predicted values from the regression model with education

$\hat{\beta }$ = estimated coefficient

Cognitive age was then modeled via multiple linear regression, with the selected neuropsychological scores as predictors and chronological age as the dependent variable, following prior work [8]. For each participant, the regression model outputs their predicted cognitive age. However, well-known bias in age modeling is the tendency for younger participants to be assigned higher ages, while older participants are assigned lower ages than their actual ages. This phenomenon is attributed to a regression to the mean problem [60,61]. This bias can be conceptualized as our best estimate for a subject about whom we have no prior information being the mean age. The predicted age bias can be corrected [62], by considering the actual participant’s age through fitting a linear model as shown in AgeML [63], thus obtaining a bias-corrected predicted cognitive age.

The final model intercept and coefficients (original scale and standardized β) are reported in Appendix A Table A1. To ensure full transparency and reproducibility, the complete training and prediction pipeline, including the model weights and scripts used for the present analyses, is publicly available at https://github.com/itellaetxe/cognitive_age_delta_jcm (accessed on 8 November 2025). Specific details on the model training can be found in the Methods section of AgeML [63]. In summary, with *y* as age, *X* as our feature set and *f*() as our pipeline, we optimize the parameters of our pipeline *f*() by means of minimizing the mean squared error (MSE) of age on CUA-V- subjects. Our pipeline consists of a feature scaler and a linear regressor, and we use a 5-fold cross-validation scheme. Finally, the Cognitive Age Delta (CAD) was defined as in Equation (2):

CAD = ŷ − y(2)

where:

ŷ = bias-corrected predicted cognitive age

y = chronological age

### 2.12. Statistical Analysis

All statistical analyses were performed in R version 4.5.1 (R Foundation for Statistical Computing, Vienna, Austria). Chronological Age and CADs distribution means across all clinical groups were compared using non-parametric Kruskal-Wallis tests. We analyzed the effect of the selected factors (modifiable and non-modifiable) with General Linear Models (GLMs) of the shape (CAD~Factor) for each clinical group (CUA-V-, CUA+, CUV+). If the factor showed a statistically significant association with the CADs within the clinical group, a sensitivity analysis was conducted adding sex, APOE4 allele load, and years of education to the model as covariates (CAD~Factor + sex + APOE4 Load + Years of Education). The latter covariate was included to consider its possible association with the factor of interest, thus isolating the effect of the factor. All input variables were standardized to allow comparing their effects. Factors were analyzed in groups to prevent multiple comparisons corrections from throwing off all possible associations, due to our modest sample size. A type I error rate of 5% (= 0.05) was set for all analyses, after correcting for multiple comparisons within each group using FDR. We analyzed these factor groups: neurological history, motor scales, cognitive symptoms, neuropsychiatric symptoms, stress and sleep questionnaires, cardiovascular parameters, cognitive reserve related factors, tobacco and alcohol consumption, physical activity and sedentarism, and adherence to Mediterranean diet. Associations between the available CSF markers and CADs were explored also using GLMs correcting for years of education, sex, and APOE4 allele load.

**Figure 2:**
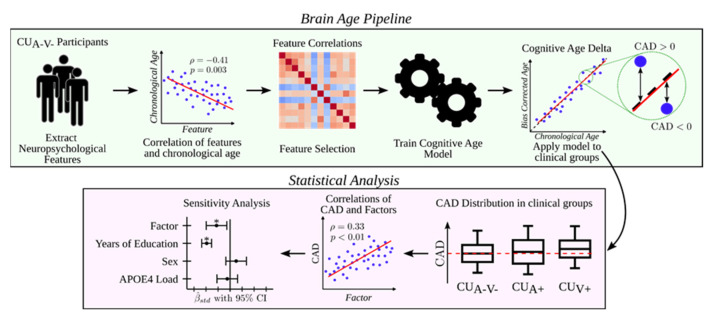
Workflow of CAD computation and subsequent statistical analyses. The figure illustrates the main processing steps used to obtain the Cognitive Age Delta (CAD) from neuropsychological data, including age bias correction, and the subsequent analyses exploring associations between CAD and clinical, biological, and lifestyle factors. * indicates significant difference (*p* < 0.05).

Figure 2 summarizes the analytical workflow, including CAD computation and subsequent statistical analyses.

## 3. Results

### 3.1. Sample Characteristics

A total of 177 CU participants were included in the analyses: 140 CUA−V−, 23 CUA+, and 14 CUV+. Two additional CUA−V− participants were excluded from the initial CU group (n = 179) due to missing data in the neuropsychological tests required for CAD computation. The three subgroups did not differ significantly in sex distribution (χ^2^ = 1.38, *p* = 0.50) or years of education (Kruskal–Wallis test, H(2) = 0.33, *p* = 0.84). However, significant age differences were observed among groups (Kruskal–Wallis test, H(2) = 14.39, *p* = 0.001), with CUA−V− participants being on average about 5 years younger than both CUA+ and CUV+ individuals. Given the mean cohort age (55–60 years), this difference—although statistically significant—is unlikely to be of major clinical relevance. The frequency of APOE ε4 carriers was higher in the CUA+ group compared to CUA−V− and CUV+ (χ^2^ = 7.69, *p* = 0.021). Median (interquartile range, IQR) and frequency (%) values are shown in Table 1.

Additionally, there were no significant differences in age, sex distribution, years of education, and MCI or APOE ε4 carrier frequency between participants who underwent lumbar puncture and those who did not (Appendix A Table A2), indicating that CSF data availability was not biased by major demographic or genetic factors.

### 3.2. CAD Distribution and Group Comparisons

**Figure 3:**
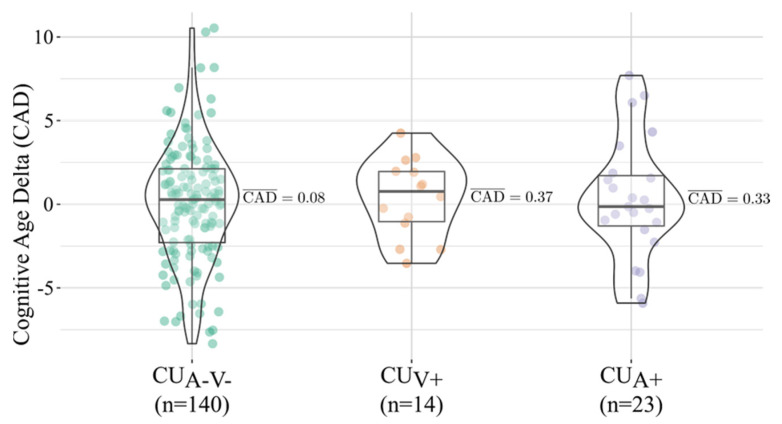
Comparison of Cognitive Age Delta (CAD) values among CU subgroups (CUA−V−, CUA+, and CUV+).

No significant differences in CAD were observed among the three CU subgroups (Kruskal–Wallis test, H(2) = 0.17, *p* = 0.91). Median (IQR) CAD values were [0.28 (−4.13, 4.69)] for CUA-V-, [−0.14 (−3.15, 2.87)] for CUA+, and [0.77 (−2.22, 3.76)] for CUV+, indicating a comparable distribution of cognitive performance relative to chronological age across groups (Figure 3).

### 3.3. Associations Between CAD and Cognitive Reserve Related Variables

**Figure 4:**
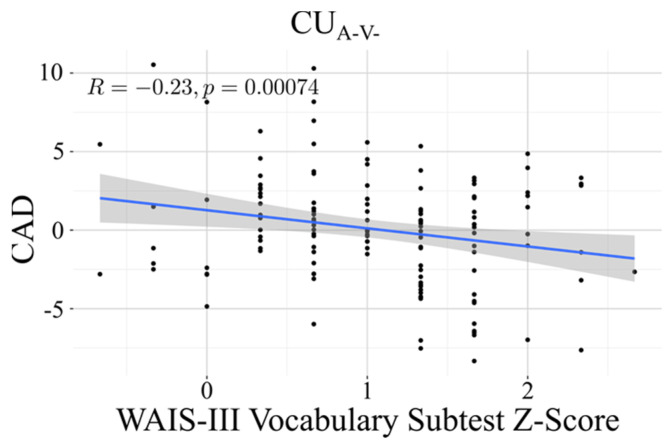
Association between Cognitive Age Delta (CAD) and WAIS-III Vocabulary z-scores in the CU_A−V−_ group. The scatterplot shows the linear relationship between CAD and Vocabulary performance in the biomarker-negative clinical group, after correcting for years of education, sex, and APOE ε4 load. The fitted regression line (solid) and the 95% confidence interval (shaded area) are displayed. Higher Vocabulary scores were associated with a lower (younger) predicted cognitive age.

In the stratified analyses, a significant association was found between CAD and the Vocabulary subtest from the WAIS-III, independent of years of education, sex, and APOE4 allele load in the CUA-V- group. This association did not survive FDR correction in the amyloid positive group, although the 95% confidence interval (CI) did not include 0 (CUA-V-: β = −1.39, *p* <0.001); CUA+: β = −2.08, *p* = 0.054). All *p*-values are reported after FDR correction. Figure 4 shows the association observed in the CUA-V- group.

In addition, within the CUA+ group, the association between the Cognitive Reserve Questionnaire and CAD also did not survive FDR correction, despite the 95% CI not including 0 (β = −2.47, *p* = 0.054). No significant associations were found for the Leisure and Productive Activities Questionnaire or for bilingualism (*p* < 0.05 post FDR correction).

### 3.4. Associations Between CAD and Lifestyle Factors

**Figure 5:**
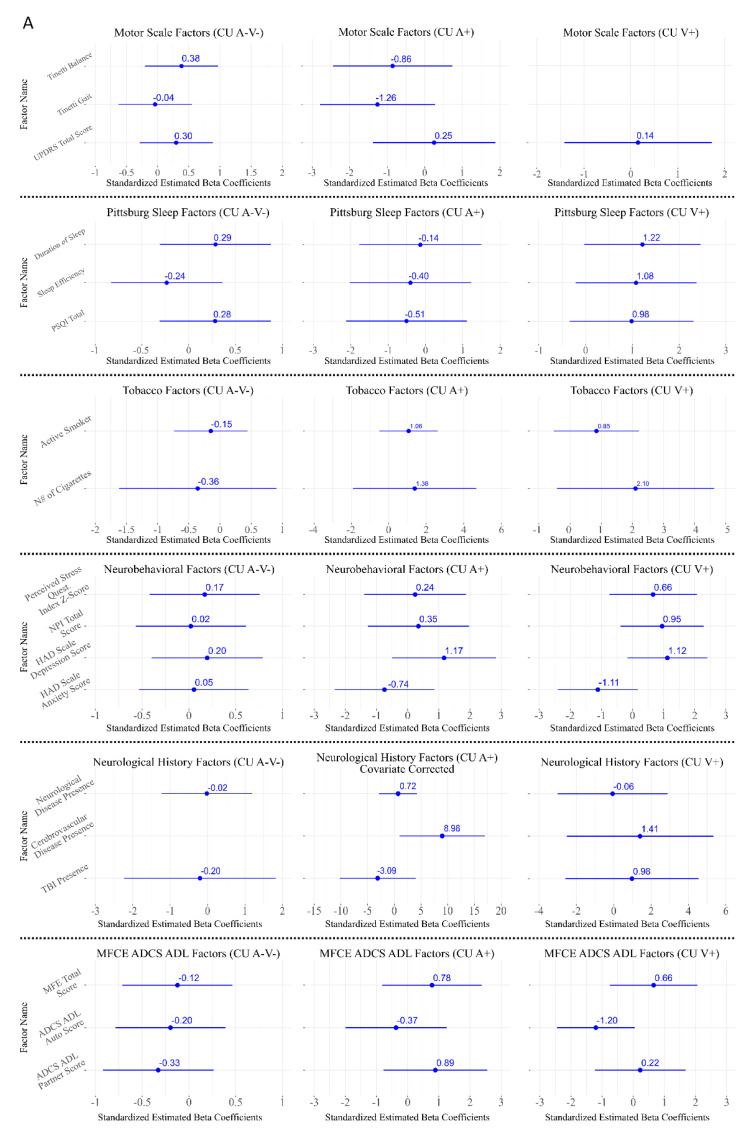

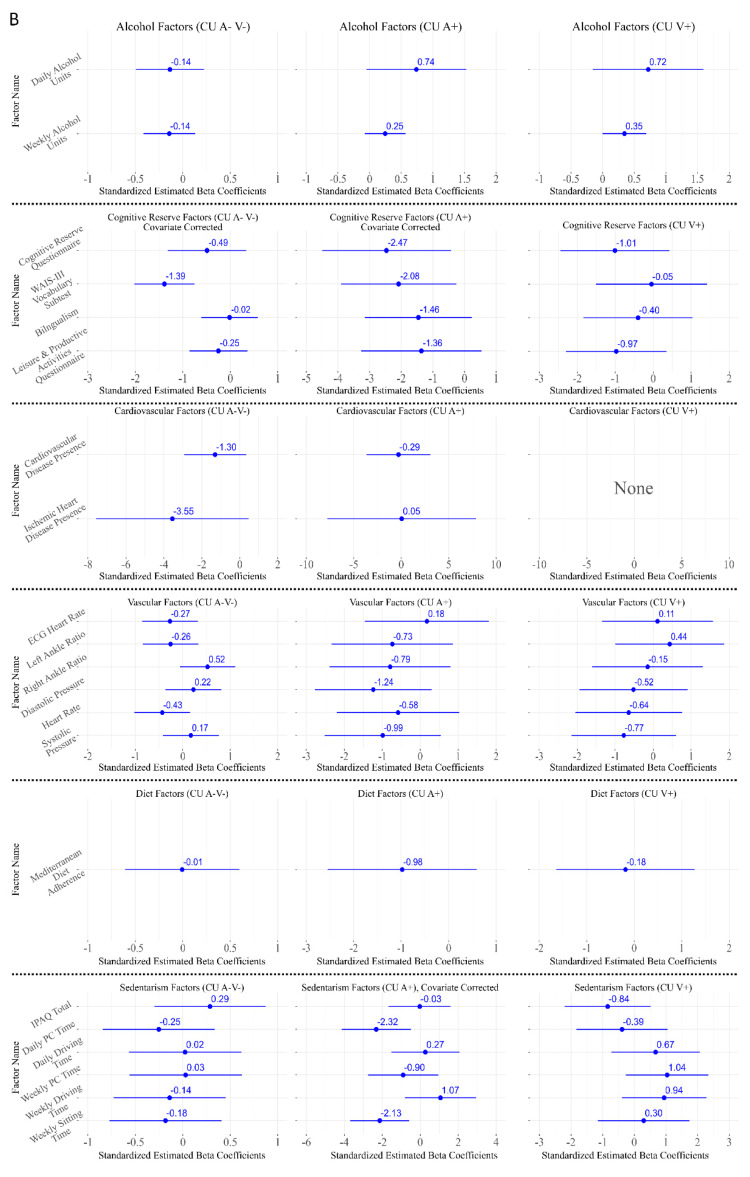
Associations between Cognitive Age Delta (CAD) and the studied factors across Cognitively Unimpaired (CU) subgroups. (**A**) Includes Motor Scales, Pittsburg Sleep Questionnaire, tobacco use, neurobehavioral symptoms, neurological history, Memory Failures of Everyday Questionnaire (MFCE) and Assessment of Instrumental Activities of Daily Living (ADCS-ADL). (**B**) Includes alcohol use, cognitive reserve, cardiovascular disease, vascular parameters, diet, and International Physical Activity Questionnaire (IPAQ) and sedentary behavior. Standardized beta coefficients (β) and their 95% confidence intervals are shown for each CU subgroup (CUA−V−, CUA+, and CUV+). If the group title includes “covariate-corrected”, the plot represents the results of the sensitivity analyses after including sex, APOE ε4 load, and years of education as covariates in the GLM. Only Cognitive Reserve– and Sedentarism–related factors remained significant after sensitivity and multiple-comparison corrections (*p* < 0.05 uncorrected; *p* < 0.05 FDR-corrected).

Regarding lifestyle-related variables, a significant association was observed between daily hours spent sitting in total and hours spent working seated from Monday to Friday at a computer and CAD only in the CUA+ group (β = −2.13, *p* = 0.046 and β = −2.32, *p* = 0.046, respectively) (Figure 5).

In contrast, no significant associations were found for physical activity, diet, tobacco and alcohol consumption in any of the groups (*p* < 0.05 post FDR correction) (Figure 5).

### 3.5. Associations Between CAD and Neurological Assessment

No significant associations were detected between CAD and clinical or neurological variables, including neurological history (any neurological disease, traumatic brain injury, or cerebrovascular disease including transit ischemic attack), motor scales (UPDRS-III and Tinetti), or subjective cognitive complaints across any subgroup categorized via a detailed neurological history (all *p* > 0.05) (Figure 5).

### 3.6. Associations Between CAD and Cardiovascular Risk

Similarly, CAD was not associated with vascular risk factors or vascular parameters, including cardiovascular disease, systolic and diastolic blood pressure, or heart rate, in any of the groups (all *p* > 0.05) (Figure 5).

### 3.7. Associations Between CAD and Neurobehavioral and Sleep-Related Factors

No significant associations were found between CAD and neurobehavioral variables, including depressive and anxiety symptoms measured with the HADS, neuropsychiatric symptoms assessed with the NPI, or perceived stress (PSQ). Perceived sleep quality, evaluated with the PSQI, was also not significantly related to CAD in any subgroup (all *p* > 0.05) (Figure 5).

### 3.8. Associations Between CAD and CSF Biomarkers

Finally, no significant associations were observed between CAD and CSF biomarker concentrations, including amyloid-β_42_ (Aβ_42_), phosphorylated tau (p-tau) or total tau (t-tau) (all *p* > 0.05).

## 4. Discussion

The present study leverages the CAD model—a scalable, individualized biomarker of functional brain aging—to investigate the influence of cognitive reserve, APOE genotype, sex, and lifestyle factors in a deeply phenotyped cohort of CU adults from the Basque Country (Northern Spain). Despite the absence of significant mean CAD differences among biomarker-defined subgroups (A−V−, A+, and V+), CAD captured differential associations with cognitive reserve and lifestyle factors, highlighting its sensitivity to early resilience mechanisms even before overt neurodegeneration.

### 4.1. The Value of Cognitive Age Delta in the Biomarker Era

Chronological age is an imprecise surrogate for biological aging, prompting the development of objective metrics such as Brain Age (a neuroimaging-derived structural metric) [64] and epigenetic clocks (DNA methylation-based cellular metrics) [65,66] to capture interindividual variability. While these biological clocks are crucial—accelerated brain or epigenetic age has been prospectively associated with worse cognitive outcomes and neuropathological burden—they primarily reflect structural integrity or cellular processes. In contrast, CAD provides a direct, low-cost, and non-invasive functional measure of age-related cognitive performance. Unlike molecular biomarkers, CAD directly captures interindividual variability in performance that likely reflects the integration of reserve, lifestyle, and subclinical pathology. This derived metric offers an immediately translatable snapshot of cognitive health that complements, rather than replaces, more resource-intensive neuroimaging or fluid biomarker assessments.

Our methodology extends previous CAD studies employing a comprehensive, multidomain neuropsychological battery [67,68] and stratifying CU participants according to both the AT(N) framework and cerebrovascular burden (V status) [10,69,70]. Prior research on cognitive age often relied on clinical diagnosis (e.g., MCI vs. CU), which overlooks the substantial impact of subclinical pathology. By focusing our baseline analyses on individuals without biomarker evidence of pathology (A−V−), we minimized confounding and enhanced the internal validity of CAD as a marker of genuine cognitive aging processes. This rigorous, biomarker-informed stratification allows testing whether determinants of cognitive aging differ across distinct biological risk profiles, an essential step toward precision prevention [11]. Furthermore, using a rich neuropsychological profile (NPS) helps mitigate potential misclassification errors that often occur with single-test screening or unidimensional markers [71,72].

### 4.2. APOE and Sex Effects

As expected, APOE ε4 carriers were more frequently A+, consistent with its role in promoting amyloid aggregation and impairing clearance [57,73]. Yet, APOE did not influence CAD, suggesting that genetic risk accelerates molecular pathology without necessarily translating into detectable functional aging differences prior to tau involvement. This may also relate to the low number of ε4 homozygotes, which limits power to detect genotype-dependent effects [59,74].

Sex differences were minimal overall, though growing evidence suggests women show enhanced resilience to amyloid-related changes until menopause, after which hormonal decline may amplify susceptibility to tau accumulation and cognitive aging [75,76]. Future CAD studies should explicitly model sex-by-pathology and sex-by-APOE interactions, as these represent critical modulators of vulnerability and resilience in aging.

### 4.3. CSF Biomarkers

Importantly, the A+ subgroup represents a very early preclinical phase (A+T−N−), in which amyloid accumulation occurs before tau deposition or neurodegeneration. According to the updated biological definitions of AD [9,10], tau aggregation is more closely linked to cognitive decline, explaining the absence of direct associations between CAD and CSF Aβ or p-tau. This supports the interpretation that CAD reflects resilience-related rather than pathology-driven variability

### 4.4. Rigorous Biomarker Definition for Vascular Pathology

A key methodological strength of this study lies in the rigorous and clinically meaningful definition of cerebrovascular pathology (V+), which was determined by the presence of severe white matter hyperintensities (Fazekas score ≥ 2) or ≥4 CMBs. This dual criterion was selected not only for its alignment with current clinical trial standards, as ≥4 CMBs represent the exclusion threshold in major anti-amyloid monoclonal antibody studies (e.g., Donanemab, Lecanemab) due to the increased risk of ARIA, but also because it reflects a threshold of clinically significant cerebrovascular frailty [51,52,53,54].

The selection of Fazekas score ≥ 2 (denoting confluent WMH) defines a high white matter hyperintensity burden, a parameter consistently associated with greater cognitive dysfunction and successful prediction of cognitive performance in aging and AD cohorts. Similarly, the consensus threshold of ≥4 CMBs defines a high-risk group. CMBs, which represent prior microscopic hemorrhages, are key imaging markers of underlying small vessel disease (SVD) and vessel wall integrity impairment, independently linked to increased risk of stroke, mortality, and cognitive deterioration. The co-occurrence of extensive WMH and/or multiple CMBs marks a state of heightened small-vessel vulnerability, associated with impaired vascular repair, endothelial dysfunction, and disrupted neurovascular coupling.

While our primary criteria focus on these two widely used markers, the V+ definition captures the broader spectrum of SVD, which includes other severe manifestations such as lacunar infarcts and cSS. By adopting high-burden thresholds for WMH and CMBs, we effectively select individuals with a clinically and biologically significant SVD burden.

The use of SWI for microbleed detection further enhances the precision of this definition. SWI provides superior sensitivity and spatial resolution for identifying small-vessel pathology, detecting substantially more CMBs than conventional T2*-weighted Gradient-Recalled Echo (GRE) sequences [77]. This methodological choice strengthens the accuracy of vascular burden classification, thereby providing a more nuanced framework for examining the interplay between vascular pathology, cognitive aging, and resilience mechanisms.

### 4.5. Differential Associations of Cognitive Reserve and Lifestyle Factors

The present study focused on the CUA−V−, CUA+, and CUV+ subgroups, deliberately omitting specific analysis of T+ groups (e.g., A+T+) due to the small sample size in these less-represented categories (n = 23 for A+, n = 14 for V+). Statistical power limitations preclude reliable inference in these combined pathology groups. Crucially, the CUA+ cohort, specifically in the absence of tau pathology (T−) or neurodegeneration (N−), aligns with the earliest conceptualization of the AD continuum, potentially capturing individuals decades before clinical onset. The ability of CAD to detect functional associations in this preclinical stage is a key contribution.

Our analysis revealed a significant negative association between Vocabulary score and CAD in the pathology-free group (CUA−V−: β^ = −1.39, *p* < 0.001), a finding that was replicated in the CUA+ group (β = −2.08, *p* = 0.054). Vocabulary performance is traditionally considered a robust indicator of verbal intelligence [38]. Beyond reflecting linguistic proficiency, it is often used as a proxy for cognitive reserve (CR) [7,78] capturing cumulative lifetime intellectual enrichment. Consistent with our results, prior work has shown that higher premorbid IQ (assessed with the Wechsler Test of Premorbid Functioning (TOPF)) is associated with a younger cognitive age estimate, even after covariate adjustment [68]. This robust inverse relationship, where richer vocabulary as an estimate of premorbid intelligence predicts a “younger” cognitive age (lower CAD), strongly supports the CR hypothesis. Within the updated conceptual framework proposed by [79], CR is defined as a brain property enabling better than expected cognitive performance given the degree of brain aging or pathology. Individuals with higher vocabulary scores may thus rely on more efficient or flexible neural networks that help maintain cognitive function despite age-related brain changes. Moreover, our results are aligned with evidence from previous studies showing that premorbid verbal ability, as indexed by reading or vocabulary tests, operates as an independent determinant of cognitive performance in both healthy individuals and those in the AD continuum [80,81]. Accordingly, vocabulary can be understood as a prototypical CR proxy, and its association with CAD supports the interpretation of CAD as a behavioral expression of functional resilience, the capacity to sustain efficient cognition despite age-related neural alterations. The persistence of this association in the CUA+ cohort is particularly relevant, suggesting that CR remains a critical factor modulating functional cognitive performance even in the presence of preclinical amyloid pathology.

In contrast, apart from a strong association with vocabulary, leisure and productive activities, the Cognitive Reserve Questionnaire, and bilingualism did not emerge as significant predictors. This may reflect the fact that lifestyle-based proxies of CR exert stronger effects on longitudinal trajectories than in cross-sectional analyses, and that the homogeneity and high educational attainment of this cohort may have limited the variability required to detect such associations. Nevertheless, previous research has consistently highlighted the role of these factors as contributors to cognitive reserve. Participation in cognitively stimulating leisure activities across the lifespan has been linked to delayed cognitive decline and reduced dementia risk [82,83], while standardized questionnaires such as the Cognitive Reserve Questionnaire capture the cumulative contribution of education, occupation, and enrichment activities to reserve [42,84]. Moreover, the protective impact of bilingualism is known to depend on factors such as proficiency, age of acquisition, and daily use [85], and recent reviews emphasize that while it may not reduce pathology incidence, bilingualism can delay symptom onset and enhance resilience to neuropathological burden [86]. Taken together, these findings suggest that CAD may be particularly sensitive to stable, crystallized proxies of reserve, such as vocabulary and educational attainment, whereas more dynamic or context-dependent indicators require longitudinal investigation to determine their contribution to functional cognitive aging.

An unexpected finding emerged in the CUA+ group, where more hours spent sitting daily was associated with a lower CAD (β^ = −2.13, *p* = 0.009). This finding appears counterintuitive, as high sedentary behavior is consistently linked to increased neurodegeneration and worse cognitive performance [87,88]. However, this paradoxical association should be interpreted cautiously within the context of our highly educated, research-volunteer cohort. The significant association was detected for both “daily hours spent sitting in total” and hours spent working seated at a computer. This suggests a potential confounding mechanism: high total sitting time in this academically privileged cohort may reflect time spent engaged in cognitively demanding, non-physical activities (e.g., computer-based intellectual work, reading) which themselves contribute positively to cognitive reserve and resilience. Indeed, recent studies have highlighted that the cognitive consequences of sedentary behavior differ according to its nature, with cognitively active sedentary behaviors showing neutral or even beneficial associations compared to passive activities such as television viewing [89,90,91].

Furthermore, it is important to note that the total sitting time assessed here includes time spent in bed at night that is not dedicated to sleeping, a metric that may correlate with underlying sleep problems, but is distinct from the formal sleep quality index (PSQI) which was non-significant. Sleep, in particular, represents a modifiable determinant tightly linked to amyloid clearance through glymphatic pathways and to vascular health [92,93]. Short sleep duration, sleep fragmentation, and untreated obstructive sleep apnea (OSA) are all associated with increased Aβ accumulation, endothelial dysfunction, and white matter damage, which may converge to accelerate cognitive aging [94].

Finally, the remaining factors investigated—including cardiovascular risk parameters, physical function, and socio-emotional status (e.g., perceived stress) —did not show significant associations with CAD in this cross-sectional, CU cohort. This absence of association contrasts with the compelling evidence from multidomain intervention trials, such as the FINGER study [17] and the CITA GO-ON trial (part of the World-Wide FINGERS network) [95], which directly target these very factors to prevent cognitive decline. The null findings in our cohort may reflect several possibilities: (a) CAD captures the functional state of resilience (CR, as evidenced by Vocabulary) but is less sensitive to the cross-sectional state of modifiable risk factors; (b) the modest sample size in the A+ and V+ subgroups limited the power to detect subtle, multifactorial effects; or (c) the impact of these factors primarily manifests over time, affecting the rate of cognitive decline rather than the baseline cross-sectional CAD score, emphasizing the necessity of longitudinal follow-up.

### 4.6. Strengths

Despite these limitations, the study presents several notable methodological strengths. Participants underwent extensive clinical and biological characterization using gold-standard biomarkers, including CSF analyses for amyloid and tau, and high-resolution 3T MRI. Critically, vascular pathology was assessed with superior diagnostic sensitivity, utilizing not only conventional Fazekas scoring but also SWI to identify cerebral microbleeds. This rigorous, multimodal approach and the strict biomarker-based classification of cognitively unimpaired individuals (A-T-N- and V-) enhance the internal validity of the findings. Fundamentally, the integration of CAD modeling offers an innovative framework for moving beyond chronological age, yielding a sensitive, scalable biomarker of functional brain aging. The added value of CAD lies in its potential for personalized medicine and prevention, enabling the early, individualized identification of modifiable determinants of cognitive resilience. This ability to track subtle deviations in cognitive performance before clinical symptoms emerge positions CAD as a highly useful outcome measure for preclinical AD clinical trials, including both pharmacological interventions and non-pharmacological lifestyle programs (such as FINGER-like trials), providing a precise and earlier metric for tracking intervention effects in high-risk populations

### 4.7. Limitations

The findings of this study must be considered within the context of several methodological constraints that should be acknowledged. First, the cross-sectional design precludes establishing causal inference regarding the directionality of the observed associations between cognitive age and lifestyle or biological factors, meaning longitudinal data will be essential to determine whether these variables truly influence the trajectory of cognitive aging or reflect reverse causation. While longitudinal follow-up data for the GAP clinical-biological cohort are available, we first prioritized a cross-sectional baseline analysis to generate preliminary hypotheses and establish the feasibility and added value of calculating CAD metric at a single time point. This metric is intended to detect subtle cognitive changes in relation to associated amyloid and/or vascular pathology, as well as exposure to modifiable and non-modifiable risk and protective factors. This decision is largely motivated by the baseline visit offering the most robust characterization of the cohort: it includes the largest sample size and the highest number of subjects with exhaustive clinical-biological phenotyping. Crucially, the baseline assessment maximizes the statistical power for our primary study focus—the cognitively unimpaired group—as subject attrition in later follow-up visits (common in similar cohorts) progressively reduces the available sample size and increases the proportion of participants with age-related cognitive impairment.

Second, participants were recruited from an ongoing research cohort rather than a representative population-based sample, which may limit the generalizability of the findings, as individuals who volunteer for biomarker-based studies often possess higher education and health literacy. Furthermore, the racial and ethnic composition of our sample was predominantly Caucasian, restricting extrapolation to more diverse populations. We must consider the potential for ceiling effects in the neuropsychological assessments, as the tests used were primarily designed for detecting mild cognitive impairment and dementia, which may reduce their sensitivity to subtle cognitive variations among cognitively unimpaired middle-aged adults. To minimize this potential confounding effect, we selected a battery of cognitive tests specifically designed to capture age- or aging-associated changes within the relevant cognitive domains. Furthermore, the use of pre-established specific cognitive composites was not initially planned, as there is currently no broad consensus regarding their optimal construction. Cognitive composites are highly context-dependent and must be tailored to several factors: the specific study group, the anticipated underlying pathology, the objective of the neuropsychological assessment (e.g., diagnosing cognitive impairment versus detecting subtle changes in cognitively unimpaired individuals), and the prior experience of the research team in their previous work.

Finally, the relatively modest sample size limits statistical power to detect small effects and precludes the exploration of potential synergistic interactions between amyloid and vascular pathology (A+V+), as well as possible associations with tau pathology.

### 4.8. Future Directions

Future research should prioritize the longitudinal validation of the CAD framework to determine its predictive value for cognitive decline with and without amyloid and vascular burden, as well as its responsiveness to preventive interventions. Indeed, from the Gipuzkoa Alzheimer Project (GAP), we have data for two additional follow-up visits over the next 6 years, following the baseline data used in this manuscript. In future works, we expect to explore specifically if (1) CAD could be a prognostic measure to detect those subjects that may convert from cognitively unimpaired to MCI, mainly in those at higher risk due to amyloid positivity and/or vascular pathology, (2) to recheck the proposed CAD assessment in a slightly older population, (3) to assess its sensitivity to detect intraindividual differences in the follow up, and (4) to specifically address even if it not a baseline, in the follow up, if exposure to modifiable risk and protective factors of dementia may have a higher impact in CAD differences. These future longitudinal analyses may help to confirm the best performing cognitive tests in middle age and early elderly population in relation to detecting subtle changes in cognitive performance about both healthy and pathological aging, and not only for diagnosis purposes of cognitive decline, and this could be the first step to create potential composite cognitive scores for this specific purpose that may facilitate more standardized assessment in different clinical contexts.

External validation in independent and more diverse cohorts is essential to confirm generalizability, ideally leveraging less invasive plasma biomarkers, such as p-tau217, which has demonstrated high diagnostic accuracy for defining amyloid positivity (A+), to enable larger-scale and more inclusive studies. Expanding CAD into a multimodal biological aging framework—for instance, integrating complementary markers such as brain age derived from MRI or epigenetic clocks based on DNA methylation—could provide a more comprehensive understanding of resilience and vulnerability mechanisms. Incorporating digital biomarkers from wearable devices and smartphone-based assessments will further enhance ecological validity and sensitivity to subtle, real-world cognitive changes. Finally, CAD holds strong translational potential as an individualized and sensitive outcome measure in both pharmacological and lifestyle-based prevention trials targeting preclinical AD and vascular dementia.

## 5. Conclusions

In summary, this study validates CAD as a sensitive and scalable index of functional brain aging that captures interindividual variability beyond traditional Alzheimer’s disease and vascular biomarkers. CAD effectively identifies mechanisms of cognitive resilience, as evidenced by the robust association between higher Vocabulary—a marker of cognitive reserve—and a younger cognitive age in both pathology-free (A−V−) and preclinical amyloid-positive (A+) participants. The absence of strong effects from amyloid or vascular pathology underscores that functional cognitive aging may be modulated earlier and more dynamically by lifelong intellectual enrichment and lifestyle factors than by static biological lesions. Although cross-sectional, these findings highlight the potential of CAD to identify individuals with preserved cognitive efficiency despite biological vulnerability. Future longitudinal and multimodal studies integrating CAD with neuroimaging, plasma biomarkers, sleep and cardiovascular profiles, and digital monitoring will be essential to delineate the temporal interplay between lifestyle, systemic health, and molecular pathology. Ultimately, this approach supports the transition toward precision prevention strategies aimed at sustaining brain maintenance and delaying the onset of cognitive impairment across the aging continuum.

## Figures and Tables

**Table 1:** Characteristics of the study participants by study subgroups.

	CUA-V- (n = 140)	CUA+ (n = 23)	CUV+ (n = 14)	*p*-Value
Age, years, median (IQR)	55 (52–60)	61 (55–66)	62 (58.25–66.25)	0.001
Sex, female, n (%)	79 (55.6%)	11 (47.8%)	6 (42.9%)	0.08
Years of education, median (IQR)	14 (12–16)	14 (10–16)	13 (11–18.25)	0.82
APOE ε4 carrier, n (%)	29 (20.7%)	11 (47.8%)	3 (21.4%)	0.02

IQR = interquartile range; n = sample size.

## Data Availability

The original data and analysis scripts used in this study are available from the authors upon request. Requests for access should be directed to the corresponding authors. The scripts used in this study can be found in https://github.com/itellaetxe/cognitive_age_delta_jcm. (accessed on 1 September 2025).

## Abbreviations

The following abbreviations are used in this manuscript:

AD	Alzheimer’s disease
ADCS-ADL	Alzheimer’s Disease Cooperative Study–Activities of Daily Living
AFAGI	Asociación de Familiares de Alzheimer y otras Demencias de Gipuzkoa
Aβ42	Amyloid beta 1–42 peptide
APOE	Apolipoprotein E
ARIA	Amyloid-Related Imaging Abnormalities
AT(N)	Amyloid, Tau, and Neurodegeneration classification framework
A+/T+/N+/V+	Positive for Amyloid/Tau/Neurodegeneration/Vascular burden
BMI	Body Mass Index
BNT	Boston Naming Test
CAD	Cognitive Age Delta
CITA	Center for Research and Memory Clinic, CITA-Alzheimer Foundation
CI	Confidence Interval
CMBs	Cerebral Microbleeds
cSS	Cortical Superficial Siderosis
CSF	Cerebrospinal Fluid
CR	Cognitive Reserve
CU	Cognitively Unimpaired
CUA−V−/	Cognitively Unimpaired Amyloid- (A-T-N-) and Vascular–
CUA+	Cognitively Unimpaired Amyloid+ (A+ and T and N+ or -) and Vascular–
CUV+	Cognitively Unimpaired Vascular+ and Amyloid- (A-T-N-)
DNA	Deoxyribonucleic Acid
ELISA	Enzyme-Linked Immunosorbent Assay
ENRICA	Estudio de Nutrición y Riesgo Cardiovascular (Spanish Study on Nutrition and Cardiovascular Risk)
FCSRT	Free and Cued Selective Reminding Test
FDR	False Discovery Rate
FLAIR	Fluid-Attenuated Inversion Recovery
GAP	Gipuzkoa Alzheimer Project
GLM	General Linear Model
GRE	Gradient-Recalled Echo
HADS	Hospital Anxiety and Depression Scale
HbA1c	Glycated Hemoglobin
HDL	High-Density Lipoprotein
IPAQ	International Physical Activity Questionnaire
IQR	Interquartile Range
IWG-3	International Working Group Criteria, 3rd Revision
JLO	Judgment of Line Orientation Test
LDL	Low-Density Lipoprotein
M@T	Memory Alteration Test
MCI	Mild Cognitive Impairment
MMSE	Mini-Mental State Examination
MRI	Magnetic Resonance Imaging
MTA	Medial Temporal Atrophy scale
NPI	Neuropsychiatric Inventory
NPS	Neuropsychological Profile
OSA	Obstructive Sleep Apnea
PCR	Polymerase Chain Reaction
p-tau	Phosphorylated tau
t-tau	Total tau
PSQ	Perceived Stress Questionnaire
PSQI	Pittsburgh Sleep Quality Index
ROCF	Rey–Osterrieth Complex Figure Test
SVD	Small Vessel Disease
SWI	Susceptibility-Weighted Imaging
STRIVE/STRIVE-2	Standards for Reporting Vascular Changes on Neuroimaging (1st and 2nd editions)
SUN	Seguimiento Universidad de Navarra
TMT-A/TMT-B	Trail Making Test Parts A and B
TOPF	Wechsler Test of Premorbid Functioning
UPDRS-III	Unified Parkinson’s Disease Rating Scale, Part III (motor section)
V	Vascular status (burden)
WAIS-III	Wechsler Adult Intelligence Scale, 3rd Edition
WMH	White Matter Hyperintensities

## Appendix A

**Table A1:** Regression coefficients (B) and standardized weights (β) of neuropsychological test variables contributing to the Cognitive Age model after bias correction.

	Standardized Coefficient (β)	Original Scale Coefficient (B)
Digit Symbol WAIS-III	−1.7630	−0.1156
Stroop Color	−0.8945	−0.0741
Stroop Word-Color	−1.1618	−0.1138
15 Object test	−0.6991	−0.4875
ROCF Recall 30 min	−1.3264	−0.2507
TMT-A	0.1797	0.0172
FCSRT Immediate Total Free Recall	−0.6966	−0.1280
TMT-B	−0.9684	0.0393
Boston naming test	−0.1727	−0.0519
Phonological verbal fluency (“p”)	−0.0218	−0.0046
FCSRT Immediate Total Recall	−0.2325	−0.0595
Semantic verbal fluency (“animals”)	0.0886	0.0150

Intercept = 96.25 (Original coefficient scale), Standardized Intercept = 55.77. Neuropsychological test scores used as predictors of chronological age in the Cognitive Age Model were adjusted for years of education prior to model training. Standardized coefficients β represent the change in age per 1 standard deviation increase in each neuropsychological test. Abbreviations: WAIS-III, Wechsler Adult Intelligence Scale, 3rd edition; ROCF, Rey–Osterrieth Complex Figure; FCSRT, Free and Cued Selective Reminding Test; TMT-A/B, Trail Making Test Part A/B.

**Table A2:** Characteristics of participants with and without CSF data.

	With CSF Data (n = 239)	Without CSF Data (n = 172)	*p*-Value
Age, years, mean ± SD	57 ± 7	57 ± 7	0.36
Sex, female, n (%)	125 (52.3%)	103 (59.9%)	0.08
Years of education, mean ± SD	14 ± 4	14 ± 4	0.71
MCI syndromic diagnosis, n (%)	39 (16.3%)	23 (13.4%)	0.25
APOE ε4 carrier, n (%)	60 (25.1%)	44 (25.6%)	0.39

Abbreviations: CSF = Cerebrospinal fluid; SD = Standard Deviation.
